# P274: Evolution of prevalence rate of hospital acquired infections after nine years of implementation of the recommendations in the blink clinic of Chu Tizi-Ouzou

**DOI:** 10.1186/2047-2994-2-S1-P274

**Published:** 2013-06-20

**Authors:** F Toudeft, A Azzam, F issiakem, F Saidi, D Haouchine, N Halli, N Bekri, MA Saadi, A Makhloufi, M Chebrek

**Affiliations:** 1 CHU of Tizi-Ouzou, Tizi-Ouzou, Algeria

## Introduction

Since the implementation of a program to fight against nosocomial infections in 2003, several actions were taken within the CHU of Tizi-Ouzou. Among the means of evaluation of this program, we note the development of 7 prevalence surveys of Nosocomial infection (NI).

## Objectives

Determine the evolution of the prevalence of NI; - Identify sites most recurrent infections to implement specific actions; - Evaluate the actions against the NI in the establishment.

## Methods

Surveys were conducted in 2003 and focused on the entire population hospitalized for periods extending over a period of 5 days during the month of October of each year of study. The collection of information is performed by a questionnaire pre-established on the basis of medical records confirmed cases of infection by culture or direct examination by the microbiology laboratory, examining use has been bedside and each bed is consulted once. The data analysis software Epi info6.

## Results

The prevalence of infection decreased from 12.06% (2003) to 6.2% (2012).

Operative urinary sites are the most recurrent infections. Surgical wards and intensive care units occupy the largest place with a rate of 33.59% IN (2003) to 14.47% (2012). As for risk factors, parenteral nutrition (31.2%), the survey nasogastric (30.4%), near the central venous and peripheral (25.6%), the catheterization (19.2%) are factors most ended up with a significant difference (p = 0.003) and an OR ranging from 4 to 9. The rate of past documentation is 66.7% (2003) to 83.3% (2010).

## Conclusion

The analysis shows that function germs for Enterobacteriaceae, E. coli is increasing over time to reduce the benefit of the genera Enterobacter and Klebsiella, Staphylococcus aureus evolves the increase to decrease in 2010 and increase to the detriment of Acinetobacter Pseudomonas. Regarding bacterial resistance, some appear to Acinetobacter and Pseudomonas. For Staphylococcus aureus, antibiotic resistance increases over time with the appearance of MRSA (BMR).

## Competing interests

None declared

